# Impact of changes in conventional risk factors induced by once-weekly GLP-1 receptor agonist exenatide on cardiovascular outcomes: an EXSCEL post hoc analysis

**DOI:** 10.1186/s12933-025-02866-7

**Published:** 2025-08-23

**Authors:** Ruth L. Coleman, Amanda I. Adler, Robert J. Mentz, Marat Fudim, Naveed Sattar, Rury R. Holman

**Affiliations:** 1 Diabetes Trials Unit, Oxford Centre for Diabetes, Endocrinology and Metabolism, Radcliffe Department of Medicine, University of Oxford, Churchill Hospital, Old Road, Headington, Oxford, OX3 7LJ UK; 2 Duke Clinical Research Institute, Duke University School of Medicine, Durham, NC USA; 3 Division of Cardiology, Duke University School of Medicine, Durham, NC USA; 4 School of Cardiovascular and Metabolic Health, University of Glasgow, Glasgow, UK

**Keywords:** Glucagon-like peptide-1 receptor agonists, Once-weekly exenatide, Cardiovascular risk factors, Cardiovascular outcomes, Model simulation, Mediation analyses

## Abstract

**Background:**

The objective of this study was to examine the degree to which conventional cardiovascular (CV) risk factor changes induced by once-weekly exenatide (EQW) might explain the placebo-controlled differences in CV outcomes observed in the Exenatide Study of Cardiovascular Event Lowering (EXSCEL).

**Methods:**

We entered participant-level risk factor values over time into a validated type 2 diabetes–specific clinical outcomes model to estimate event rates, and compared simulated with observed relative risk changes in EXSCEL. We performed simulations for each participant to minimize uncertainty and to optimize confidence interval precision around risk point estimates. Six outcomes were examined: major adverse CV event (MACE), all-cause mortality (ACM), CV death, fatal or nonfatal myocardial infarction (MI), fatal or nonfatal stroke, and hospitalization for heart failure (hHF). We also performed a mediation analysis using Cox regression models to evaluate potential key mediators for ACM.

**Results:**

Model simulations explained only modest proportions of the observed relative risk reductions for MACE (29%), ACM (15%), CV death (18%), and stroke (29%), but greater proportions for hHF (67%) and MI (200%). Mediation analysis suggested that baseline-to-6 or 12-month changes in HbA_1c_, blood pressure, heart rate, low-density lipoprotein cholesterol, triglycerides, and weight did not mediate the EQW effect on ACM.

**Conclusions:**

These model simulations explain only a modest proportion of the impact of observed EQW-induced changes in conventional CV risk factors on EXSCEL outcomes, apart from hHF and MI. Up to 1-year changes in conventional risk factors did not mediate the observed ACM risk reduction.

**Graphical abstract:**

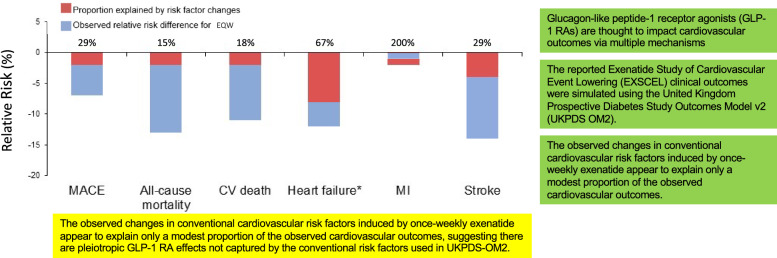

**Supplementary Information:**

The online version contains supplementary material available at 10.1186/s12933-025-02866-7.

## Research insights

**What is currently known about this topic?**

- Glucagon-like peptide-1 receptor agonists (GLP-1 RAs) affect cardiovascular (CV) outcomes via multiple mechanisms. GLP-1 RA therapies have been shown to have beneficial effects on CV disease, mortality, and kidney outcomes in people with type 2 diabetes.

**What is the key research question?**

- To what degree do the observed changes in modifiable CV risk factors induced by once-weekly use of the GLP-1 RA exenatide explain observed differences in CV event rates between the drug and placebo arms of the Exenatide Study of Cardiovascular Event Lowering (EXSCEL)?

**What is new?**

- Simulation and mediation analyses of EXSCEL both suggested observed changes in conventional CV risk factors induced by once-weekly exenatide explain only a modest proportion of the observed CV outcomes.

**How might this study influence clinical practice?**

- The results of this study could be used to adjust risk estimates derived from prediction models in people with type 2 diabetes treated with GLP-1 RAs.

## Introduction

Glucagon-like peptide-1 receptor agonists (GLP-1 RAs) have become established as blood glucose-lowering therapies that should be considered in people with type 2 diabetes if glycemic targets are not met with lifestyle measures and metformin [1], especially in those with, or at high risk of, cardiovascular (CV) disease [2]. GLP-1 RA treatment has been shown to have beneficial effects on CV disease, mortality, and kidney outcomes in people with type 2 diabetes [3, 4].

In people with type 2 diabetes, exenatide, a GLP-1 RA, reduces blood glucose levels and can lead to modest reductions in body weight, blood pressure, and lipid levels [5]. The Exenatide Study of Cardiovascular Event Lowering (EXSCEL) was a randomized event-driven trial of once-weekly exenatide (EQW) [6]. The study found that after a median 3.2 years of follow-up, participants randomized to EQW had a numerically lower incidence of major adverse CV events (MACE) and a nominally significant reduction in all-cause mortality (ACM) [7].

We sought to examine the degree to which EQW-induced changes in conventional CV risk factors might explain the observed differences in EQW and placebo event rates using model simulation and mediation analyses.

## Methods

EXSCEL was a multinational, double-blind, placebo-controlled, randomized trial evaluating the impact of EQW on CV outcomes in patients with type 2 diabetes [6, 7]. EXSCEL enrolled participants with a broad range of CV risk at 687 sites in 35 countries between June 2010 and September 2015. Eligible participants were adults with type 2 diabetes (defined as a glycated hemoglobin [HbA_1c_] concentration of 6.5% to 10.0% [48 to 96 mmol/mol]) receiving up to three oral glucose-lowering agents or receiving insulin, either alone or in combination with up to two oral glucose-lowering agents. The study was designed to enroll approximately 70% of patients with a history of a major manifestation of coronary artery disease, cerebrovascular disease or peripheral arterial disease, and 30% without known CV disease. Exclusion criteria included end-stage kidney disease or an estimated glomerular filtration rate (eGFR) of less than 30 ml/min/1.73 m^2^, high risk for medullary thyroid carcinoma, previous use of GLP-1 RA, baseline calcitonin level of > 40 ng/L, or at least two severe hypoglycemic episodes within the preceding year.

EXSCEL evaluated the effect of EQW 2 mg versus placebo when added to usual care in 14,752 participants (73.1% with and 26.9% without previous CV disease). The trial protocol was approved by the ethics committee at each participating site, and all patients provided written informed consent.

### Model simulation

We performed simulations for the primary 3-point MACE composite endpoint (CV death, nonfatal myocardial infarction [MI] or nonfatal stroke) and 5 secondary endpoints: ACM, CV death, first occurrence of a fatal or nonfatal MI, first occurrence of a fatal or nonfatal stroke, and first occurrence of hospitalization for heart failure (hHF) in individuals with no history of heart failure.

We used the United Kingdom Prospective Diabetes Study (UKPDS) Outcomes Model Version 2.1 (UKPDS-OM2) to simulate the impact of observed risk factor changes over time. UKPDS-OM2 is a second-generation lifetime-simulation model for people with type 2 diabetes that simulates single as well as composite endpoints using knowledge of previous clinical events and changes in risk factor values [8]. It predicts heart failure (based on adjudicated events) but not specifically hHF as recorded in the EXSCEL trial. UKPDS-OM2 was constructed using person-level data from the 20-year UKPDS trial and its 10-year observational follow-up. The model’s equations are based on a median 17.6 years’ follow-up with up to 89,760 person-years of data. It has been validated internally over a 25-year time horizon [8], evaluated externally [9–13], and used to estimate the likely impact of observed changes in established CV risk factors induced by empagliflozin, a sodium-glucose cotransporter-2 inhibitor (SGLT2i), on key outcomes in the EMPA-REG OUTCOME trial [14]. Missing data at baseline for modifiable risk factors were assigned the population average. We calculated event rates separately for those participants assigned to EQW and to placebo, with the two simulated rates presented as relative risks.

### Mediation analyses

The 85 participants who died prior to follow-up measurements (39 EQW, 46 placebo) were excluded from the mediation analysis, while those with missing data at baseline were assigned study population averages. Missing data at 6 and 12 months were calculated by linear interpolation (where data were available). A complete case sensitivity analysis was performed to evaluate the assumption that baseline data were missing at random.

We examined just ACM, as this was the only outcome with a statistically significant (*p* < 0.05) EQW risk reduction, compared with placebo (hazard ratio 0.86, 95% confidence interval 0.77 − 0.97) [7]. We used a causal mediation analysis (CMA) approach [15] to estimate the degree to which the effect of one or more of the CV risk factors affected by EQW could statistically account for its effect on ACM. A mediator is a variable that helps explain the relationship between a predictor and outcome variable. In other words, a predictor variable X could predict M, a mediator variable, which in turn predicts the outcome variable Y [16]. In a randomized trial, the key predictor, X, is the treatment to which an individual is randomized. Indirect effects work through mediators M_1_, M_2_, etc., whereas direct effects work through other mechanisms [17].

We performed CMA using Cox regression models, after testing proportional hazard assumptions, to evaluate potential ACM mediators using a counterfactual framework. CMA provides causal estimates of the total effect (TE) and the natural indirect effect (NIE). The TE is the overall difference between everyone being assigned EQW and the counterfactual of everyone being assigned placebo. The NIE is the estimate of the effect of the treatment via the mediator, and the proportion mediated can be calculated as the total excess risk / excess risk due to NIE. Risk factors considered included HbA_1c_, systolic blood pressure (SBP), diastolic blood pressure (DBP), heart rate, low-density lipoprotein cholesterol (LDL-C), high-density lipoprotein cholesterol (HDL-C), triglycerides, eGFR, body weight, and body mass index (BMI). We examined the effect of changes from baseline to 6 and to 12 months for all risk factors that differed by allocated treatment on ACM. We compared Cox regression models adjusted only for treatment allocation (intention-to-treat) with those adjusted for treatment allocation, baseline values, and changes in risk factor values from baseline to 6 and to 12 months. The presence of mediation was assessed as a proportion, with *p* < 0.05 considered statistically significant. All analyses were performed using SAS software v9.4.

## Results

All 14,752 EXSCEL trial participants were included in these analyses. Their baseline clinical characteristics are listed in Table S1 in the Supplementary Appendix, with baseline risk factor values and their changes over 6 and 12 months shown in Table 1. At 6 months, changes in risk factor values all differed significantly for EQW, compared with placebo, except for HDL-C and eGFR. Statistically significant differences remained at 12 months for HbA_1c_, SBP, heart rate, body weight, and BMI.

**Table 1:** Changes in risk factor values from baseline to 6 months and to 12 months

	Mean (SD) at baseline			Adjusted mean difference in change from baseline for EQW vs. placebo	
Variable	Exenatide (n = 7356)	Placebo (n = 7396)	Difference	6 months	12 months
HbA_1c_ (%)	8.1 (1.0)	8.1 (1.0)	0.0	− 0.7, *p* < 0.001	− 0.4, *p* < 0.001
HbA_1c_ (mmol/mol)	65 (8)	65 (8)	0.0	− 0.7, *p* < 0.001	− 0.4, *p* < 0.001
SBP (mmHg)	136 (17)	135 (17)	− 1.0	− 1.2, *p* < 0.001	− 2.0, *p* < 0.001
DBP (mmHg)	78 (10)	78 (10)	0	0.4, *p* = 0.020	0.2, *p* = 0.56
Heart rate (bpm)	73 (11)	73 (10)	0	3.0, *p* < 0.001	2.3, *p* < 0.001
LDL-C (mmol/L)	2.4 (0.9)	2.4 (0.9)	0.0	− 0.05, *p* < 0.001	− 0.02, *p* = 0.060
HDL-C (mmol/L)	1.13 (0.34)	1.13 (0.34)	0.00	− 0.01, *p* = 0.053	0.00, *p* = 0.94
Triglycerides (mmol/L)*	1.73 (1.71, 1.75)	1.74 (1.72, 1.76)	0.01	− 0.05, *p* = 0.002	0.02, *p* = 0.40
eGFR (mL/min/1.73 m^2^)	78 (24)	79 (24)	1	− 0.2, *p* = 0.78	0.3, *p* = 0.39
Body weight (kg)	92 (22)	92 nn(21)	0	− 1.0, *p* < 0.001	− 1.4, *p* < 0.001
BMI (kg/m2)	32.7 (6.5)	32.6 (6.3)	− 0.1	− 0.4, *p* < 0.001	− 0.5, *p* < 0.001

^*^Geometric mean ± 1 SD

EQW: once-weekly exenatide, HbA_1c_: glycated hemoglobin, SBP: systolic blood pressure, DBP: diastolic blood pressure, Bpm: beats per minute, LDL-C: low-density lipoprotein cholesterol, HDL-C: high-density lipoprotein cholesterol, eGFR: estimated glomerular filtration rate, BMI: body mass index

### Simulation analyses

Observed and simulated probabilities of time to first event in the placebo group showed good agreement for all 6 outcomes, but with underpredictions for MACE and overpredictions for ACM (Figure S1 in Supplementary Appendix). The numbers of observed and simulated events, and relative risks by treatment allocation, are shown in Table 2. In the placebo group, simulated event rates were similar to those observed for CV death (6.0% simulated vs. 5.2% observed), MI (5.1% vs. 6.7%), stroke (2.5% vs. 2.9%) and heart failure (1.3% vs. 1.2%), but lower for MACE (8.2% vs. 12.2%) and higher for ACM (12.4% vs. 7.9%). In the EQW group, simulated event rates were also similar for CV death (5.9% vs. 4.6%), MI (5.0% vs. 6.6%), stroke (2.4% vs. 2.5%) and heart failure (1.3% vs. 1.0%) but again lower for MACE (8.0% vs. 11.4%) and higher for ACM (12.2% vs. 6.9%).

**Table 2:** Observed and simulated event rates, with corresponding relative risks by treatment allocation

Event	EQW events (n = 7356)		Placebo events (n = 7396)		Observed relative risk (95% CI)	Simulated relative risk (95% CI)
	Observed	Simulated	Observed	Simulated		
Major adverse cardiovascular event	839 (11.4%)	589 (8.0%)	905 (12.2%)	603 (8.2%)	0.93 (0.83–1.03)	0.98 (0.88–1.10)
All-cause mortality	507 (6.9%)	898 (12.2%)	584 (7.9%)	920 (12.4%)	0.87 (0.75–1.00)	0.98 (0.90–1.07)
Cardiovascular death	340 (4.6%)	437 (5.9%)	383 (5.2%)	448 (6.0%)	0.89 (0.74–1.04)	0.98 (0.86–1.11)
Myocardial infarction	483 (6.6%)	369 (5.0%)	493 (6.7%)	378 (5.1%)	0.99 (0.86–1.11)	0.98 (0.85–1.13)
Stroke	187 (2.5%)	177 (2.4%)	218 (2.9%)	185 (2.5%)	0.86 (0.66–1.06)	0.96 (0.78–1.18)
Heart failure*	129 (1.0%)	99 (1.3%)	144 (1.2%)	107 (1.3%)	0.88 (0.70–1.12)	0.92 (0.70–1.21)

EQW: once-weekly exenatide

^*^Only observed/simulated in those with no prior heart failure (n = 6,194 and 6,168 for EQW and placebo, respectively)

**Fig. 1:**
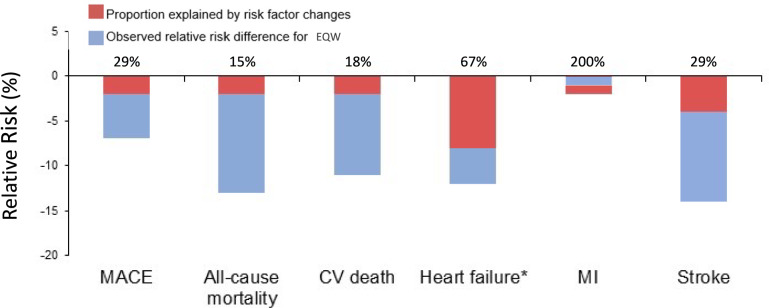
Observed placebo-adjusted relative risks for 6 EXSCEL outcomes, and proportions explained by simulation for each outcome. EQW: once-weekly exenatide, MACE: major adverse cardiovascular event, CV: cardiovascular, MI: myocardial infarction. *Only observed/simulated in those with no prior heart failure (n = 6,194 and 6,168 for exenatide and placebo, respectively)

Comparing the simulated and observed relative risks for each outcome suggested that the observed changes in CV risk factor levels during the EXSCEL trial appeared to contribute mostly to only modest proportions of the impact of EQW on the observed outcomes (Table 2, Fig. 1). The proportions of the observed relative risk reductions seen with EQW explained potentially by the combined impact of differential changes in conventional CV risk factors were 29% for MACE (2% of 7%), 15% for ACM (2% of 13%), 18% for CV death (2% of 11%), 67% for hHF (8% of 12%), 200% for MI (2% rather than 1%), and 29% for stroke (4% of 14%).

### Mediation analyses

The strongest mediator identified for ACM at 6 months was the change from baseline to 6 months for HbA_1c_, which nominally mediated the treatment effect by 10.7% (Table 3). All other risk factors accounted for < 10.0% mediation, and none were identified as statistically significant. Changes in risk factors from baseline to 12 months did not mediate the treatment effect.

**Table 3:** Causal mediation analysis of all-cause mortality risk for EQW compared with placebo

	Mediator 6-month change from baseline				Mediator 12-month change from baseline			
	HR (95% CI) total effects	Excess HR (95% CI) due to natural indirect effects	Percentage mediation	*p*-value	HR (95% CI) total effects	Excess HR (95% CI) due to natural indirect effects	Percentage mediation	*p*-value
HbA1c	0.87 (0.77–0.98)	− 0.01 (− 0.03–0.00)	10.7	0.15	0.87 (0.75–0.98)	0.00 (− 0.01–0.01)	1.3	0.79
SBP	0.87 (0.76–0.98)	0.00 (0.00–0.00)	− 0.6	0.63	0.86 (0.75–0.97)	0.00 (0.00–0.01)	− 1.1	0.61
DBP	0.88 (0.77–0.99)	0.00 (0.00–0.00)	− 0.5	0.45				
Heart rate	0.88 (0.77–0.99)	0.00 (− 0.01–0.01)	0.9	0.85	0.86 (0.75–0.98)	0.00 (− 0.01–0.01)	1.2	0.75
LDL-C	0.86 (0.75–0.98)	0.00 (0.00–0.00)	− 1.4	0.40				
Triglycerides	0.87 (0.76–0.98)	0.00 (0.00–0.00)	1.0	0.44				
Body weight	0.87 (0.76–0.98)	0.00 (0.00–0.00)	− 0.6	0.70	0.85 (0.74–0.97)	0.01 (0.00–0.02)	− 5.9	0.16
BMI	0.87 (0.76–0.98)	0.00 (0.00–0.00)	− 0.7	0.67	0.85 (0.74–0.97)	0.01 (0.00–0.02)	− 6.7	0.15

EQW: once-weekly exenatide, HR: hazard ratio, SBP: systolic blood pressure, DBP: diastolic blood pressure, LDL-C: low-density lipoprotein cholesterol, BMI: body mass index. All models adjusted for age, sex and duration of diabetes

Total Effects: the average treatment effect if everyone were treated with EQW compared to if everyone were on placebo

Natural Indirect Effects: the average change in the outcome for those treated with EQW if the mediator were changed to what it would be on placebo

## Discussion

The EQW-associated changes in modifiable risk factors observed in the EXSCEL trial appear to explain the majority of the simulated differences seen for MI and heart failure, but contribute to less than a third of the observed risk reductions seen for MACE, ACM, CV death, and stroke events. Our findings suggest that EQW may have only a modest effect on atherosclerotic-mediated outcomes over a median 3.2 years’ follow-up and that alternative mechanisms need to be explored that can further explain the magnitude of the observed risk changes in the EXSCEL trial. GLP-1 RA pleiotropic effects not captured by the conventional risk factors used in UKPDS-OM2 may include anti-inflammatory effects [18], improved endothelial function [19], and beneficial effects on cardiac structure or function [20, 21].

Causal mediation analysis did not identify any 6-month or 1-year changes in risk factors as mediators for the observed effect for ACM. Importantly, heart rate elevation did not mediate the effect of EQW on ACM, suggesting that potentially detrimental effects related to heart rate elevation are counter-balanced by an otherwise favorable metabolic profile with improved glycemia, body weight, and blood pressure. GLP-1 RA mediation analyses conducted by others have suggested HbA_1c_ and urinary-albumin-to-creatinine ratio (UACR) partly mediate the beneficial effects of dulaglutide on MACE outcomes [22], and that HbA_1c_ and SBP may moderately mediate the kidney benefits of liraglutide and semaglutide [23]. An exploratory mediation analysis of the LEADER (Liraglutide Effect and Action in Diabetes: Evaluation of Cardiovascular Outcome Results) trial identified HbA_1c_ and, to a lesser extent, UACR as potential mediators of the CV effects of liraglutide [24]. In contrast, in patients with type 2 diabetes and established CV disease treated with the SGLT2i empagliflozin, changes in hematocrit and hemoglobin were the most important mediators for the reduction in hHF and death from heart failure [25].

The differences seen in the contribution of risk factors between our simulation modelling and mediation analyses may be explained by the UKPDS-OM2 death equations not including a direct measure of HbA_1c_ as a risk factor. However, HbA_1c_ is a risk factor for both MI and stroke in the model, which in turn increase the risk of death occurring in the same year.

Limitations of these analyses include the UKPDS-OM2 poor prediction of absolute ACM event rates, with simulated rates more than 150% of those observed, although relative ACM risk did reflect the difference seen between treatment groups. One possible explanation for this overestimation may be in the differences between the population used to develop the model and the EXSCEL trial. Model recalibration may be sufficient to address this, but it could contribute to the lower estimates for the primary endpoint with non-CV death as a competing risk. Some missing data are another limitation. Lastly this is a post-hoc analysis and the potential for unmeasured mediator-outcome confounding cannot be excluded.

## Conclusions

Our simulation showed that the EXSCEL trial results can in part be attributed to EQW-induced changes in conventional CV risk factors. The observed greater benefits in outcomes may reflect more pleiotropic GLP-1 RA mechanisms, and to factors not measured in the EXSCEL study. The differences seen in the simulated and observed event rates in this study could be used to adjust risk estimates derived from prediction models, such as UKPDS-OM2, in people with type 2 diabetes treated with GLP-1 RAs.

## Supplementary Information

Additional file 1

## Data Availability

Requests for access to EXSCEL study data should be submitted via https://vivli.org/members/enquiries-about-studies-not-listed-on-the-vivli-platform.

## Abbreviations

ACM: All-cause mortality
CV: Cardiovascular
EQW: Once-weekly exenatide
EXSCEL: Exenatide study of cardiovascular event lowering
hHF: Hospitalization for heart failure
LDL-C: Low-density lipoprotein cholesterol
MACE: Major adverse CV event
MI: Myocardial infarction
UKPDS-OM2: United Kingdom Prospective Diabetes Study Outcomes Model Version 2.1
