# Supplementary material for: Impact of changes in conventional risk factors induced by once-weekly GLP-1 receptor agonist exenatide on cardiovascular outcomes: an EXSCEL post hoc analysis
Source: Cardiovasc Diabetol. 2025 Aug 23;24:347. doi: 10.1186/s12933-025-02866-7 (PMC12374271; doi:10.1186/s12933-025-02866-7)

Supplementary Appendix

Supplement to: Ruth L. Coleman, Amanda I. Adler, Robert J. Mentz, Marat Fudim, Naveed Sattar, and Rury R. Holman. Impact of Changes in Conventional Risk Factors Induced by Once-Weekly GLP-1 Receptor Agonist Exenatide on Cardiovascular Outcomes: An EXSCEL Post Hoc Analysis

### Table S1. EXSCEL participant baseline clinical characteristics.

| **Characteristic** | **All**  **N=14,752** | **Prior cardiovascular disease**  **N=10,782** | **No prior cardiovascular disease**  **N=3,970** |
| --- | --- | --- | --- |
| Age (years) | 63 (57–69) | 59.4 (52.5–65.7) | 63.9 (57.9–69.7) |
| Female sex | 5603 (38.0%) | 3580 (33.2%) | 2023 (51.0%) |
| Race |  |  |  |
| Caucasian | 11175 (75.8%) | 8134 (75.5%) | 3041 (76.6%) |
| Asian | 1452 (9.8%) | 1125 (10.4%) | 327 (8.2%) |
| Hispanic | 1134 (7.7%) | 803 (7.4%) | 331 (8.3%) |
| Black | 878 (6.0%) | 627 (5.8%) | 251 (6.3%) |
| Indian or Alaska Native | 73 (0.5%) | 63 (0.6%) | 10 (0.3%) |
| Pacific Islander or Hawaiian | 35 (0.2%) | 26 (0.2%) | 9 (0.2%) |
| Body mass index (kg/m^2^) | 31.8 (28.2–36.2) | 31.4 (28.0–35.6) | 33.0 (29.1–37.9) |
| Systolic blood pressure (mmHg) | 135 (124–145) | 135 (124–146) | 134 (124–144) |
| Diastolic blood pressure (mmHg) | 80 (70–85) | 79 (70–84) | 80 (74–86) |
| Heart rate (bpm) | 72 (66–80) | 72 (64–79) | 75 (68–81) |
| HbA_1c_ (%) | 8.0 (7.3–8.9) | 8.0 (7.3–8.8) | 8.0 (7.4–9.8) |
| Hemoglobin (g/dl) | 13.8 (12.8–14.8) | 13.8 (12.8–14.8) | 13.9 (12.9–14.8) |
| Cigarette smoking |  |  |  |
| Never smoked | 7233 (49.0%) | 4870 (45.2%) | 2363 (59.5%) |
| Prior smoker | 5791 (39.3%) | 4645 (43.1%) | 1146 (28.9%) |
| Current smoker | 1721 (11.7%) | 1260 (11.7%) | 461 (11.6%) |
| eGFR (mL/min/1.73 m^2^) | 79 (64–96) | 74 (60–90) | 82 (67–98) |
| Albumin:creatinine ratio (mg/mol) | 2.1 (0.8–7.0) | 2.4 (0.9–7.8) | 1.5 (0.6–4.3) |
| Total cholesterol (mmol/L) | 4.3 (3.6–5.2) | 4.6 (3.9–5.5) | 4.1 (3.5–5.1) |
| LDL cholesterol (mmol/L) | 2.3 (1.7–3.0) | 2.2 (1.7–2.9) | 2.5 (1.9–3.2) |
| HDL cholesterol (mmol/L) | 1.09 (0.91–1.29) | 1.06 (0.90–1.27) | 1.13 (0.96–1.34) |
| Triglycerides (mmol/L) | 1.8 (1.3–2.6) | 1.8 (1.3–2.6) | 1.8 (1.3–2.6) |
| Duration of diabetes (years) | 12 (7–18) | 10 (6–15) | 12 (7–19) |
| Atrial fibrillation | 999 (6.8%) | 820 (7.6%) | 179 (4.5%) |
| History of cardiovascular disease |  |  |  |
| Myocardial infarction | 4679 (31.7%) | 4657 (43.2%) | 22 (0.6%) |
| Stroke | 2450 (16.6%) | 2411 (22.4%) | 39 (1.0%) |
| Peripheral arterial disease | 2624 (17.8%) | 2541 (23.6%) | 83 (2.1%) |
| Heart failure | 2389 (16.2%) | 2067 (19.2%) | 322 (8.1%) |

Data are median and interquartile range or N(%).

**Figure S1.**

Observed and simulated probabilities of time to first event in the placebo and EQW groups. Placebo: A) MACE, B) ACM, C) CVD, D) MI, E) Stroke, F) Heart failure. EQW: G) MACE, H) ACM, I) CVD, J) MI, K) Stroke, L) Heart failure. Solid diamonds denote simulated point estimates. Shaded area denotes 95% confidence interval for the observed values.


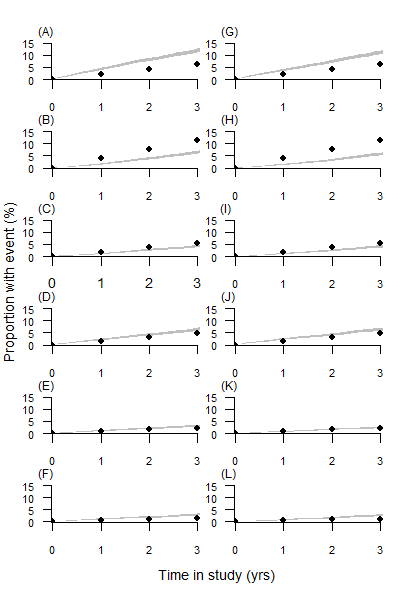

Supplement: Supplementary file 1 — Additional file 1 [file 12933_2025_2866_MOESM1_ESM.docx]
